# Physical and psychosomatic health outcomes in people bereaved by suicide compared to people bereaved by other modes of death: a systematic review

**DOI:** 10.1186/s12889-017-4930-3

**Published:** 2017-12-12

**Authors:** Ailbhe Spillane, Celine Larkin, Paul Corcoran, Karen Matvienko-Sikar, Fiona Riordan, Ella Arensman

**Affiliations:** 1 School of Public Health, University College Cork, Western Gateway Building, Cork, Ireland; 2 National Suicide Research Foundation, University College Cork, Western Gateway Building, Room 4.28, Cork, Ireland; 3 Department of Emergency Medicine, University of Massachusetts Medical School, Worcester, MA USA

**Keywords:** Suicide, Bereavement, Morbidity, Systematic review, Physical health, Psychosomatic health

## Abstract

**Background:**

Little research has been conducted into the physical health implications of suicide bereavement compared to other causes of death. There is some evidence that suicide bereaved parents have higher morbidity, particularly in terms of chronic illness. This systematic review aims to examine the physical and psychosomatic morbidities of people bereaved by a family member’s suicide and compare them with family members bereaved by other modes of death.

**Methods:**

MEDLINE, EMBASE, CINAHL, and PsycINFO were searched from 1985 to February 2016. The search was re-run in March 2017. Peer-reviewed English language articles comparing suicide-bereaved family members to non-suicide bereaved family members on measures of physical or psychosomatic health were eligible for inclusion. Cohort, cross-sectional, case-control and cohort-based register studies were eligible for inclusion. A modified version of the Newcastle Ottawa Scale was used for quality assessment. Results were synthesised using narrative synthesis.

**Results:**

The literature search located 24 studies which met the inclusion criteria. Seven studies found statistically significant associations between physical health and suicide bereavement. Five of the studies found that suicide-bereaved family members were more likely to experience pain, more physical illnesses and poorer general health. They were also at increased risk of cardiovascular disease, hypertension, diabetes and chronic obstructive pulmonary disease. In contrast, another study in Denmark found that those bereaved by suicide had a lower risk of a number of physical health disorders, including cancers, diabetes, cardiovascular and chronic lower respiratory tract disorders compared to those bereaved by other causes of death. Additionally, a further study conducted in the United States found that suicide-bereaved children visited a GP less frequently than non-suicide bereaved children.

**Conclusions:**

Review findings are relevant for clinicians working with people bereaved by suicide as they highlight that such clients are at increased risk of several adverse physical health outcomes. Future research should examine health risk behaviours of suicide-bereaved and non-suicide bereaved family members as they may confound the association between exposure and outcome.

**Trial Registrations:**

The review protocol has been registered on PROSPERO, registration number CRD42016030007.

**Electronic supplementary material:**

The online version of this article (10.1186/s12889-017-4930-3) contains supplementary material, which is available to authorized users.

## Background

Bereavement is a significant stressor that can initiate or compound existing mental and physical disorders [1]. Grief is a reaction to bereavement, encompassing thoughts, feelings, behaviours and physiological changes which may fluctuate and change in intensity over time [2]. Over 800,000 people die by suicide worldwide every year, leading to an estimated 45–500 million people experiencing suicide bereavement annually [3, 4]. Suicide has an emotional impact on those bereaved but it also has a societal impact, in terms of economic effects [5]. While a number of studies have considered the economic impact of suicide [6–8], it is important to understand the effects of suicide bereavement in terms of survivors’ physical and psychosomatic health.

There have been mixed results regarding how suicide bereavement differs from other forms of bereavement [9, 10]. A seminal review [10] posited that suicide bereavement can be differentiated by three over-arching themes. Firstly, the qualitative aspects of grief may be different, with those bereaved by suicide experiencing higher levels of guilt, blame, responsibility and rejection. Secondly, social processes may differ for those bereaved by suicide, where they feel more isolated or stigmatized due to their loved one’s suicide. Finally, a pre-existing dysfunctional family environment may have contributed to the development of suicidal thoughts and behaviour in the deceased. This dysfunction coupled with the suicide may contribute to the occurrence of psychiatric conditions amongst the surviving family members.

Also, people bereaved by suicide are at increased risk of engaging in suicidal behaviour themselves [11]. Researchers have put forward several explanations as to why those bereaved by suicide are at particular risk of suicidal behaviour. Firstly, they have established that suicidal behaviour is familial and may be partly explained by genetics [12, 13]. Research indicates that proband suicide attempt increased the odds of offspring suicide attempt by nearly 5-fold, when controlling for a number of factors including baseline history of suicide attempt [12]. Some research suggests that the intrafamilial transmission of impulsive aggression, childhood maltreatment and mood disorder may be possible mediators [12]. However, the exact mechanism underlying this genetic transmission is still unclear.

In addition, social stigma and blame represents a significant challenge for those bereaved by suicide, which may motivate some families to conceal the cause of death [14, 15]. People bereaved by suicide are at increased risk of suicide, depression, substance abuse, complicated grief and feelings of shame and guilt [10, 16–18]. They are also at increased risk of negative physical outcomes, including cardiovascular disease (CVD), chronic obstructive pulmonary disease (COPD), hypertension, diabetes and pancreatic cancer [19, 20]. While it is important to consider such specific physical health conditions, it may take many years for such conditions to develop. Therefore, it is also critical to consider somatic and psychosomatic symptoms and complaints which may be more likely to be present in the short-term following bereavement [21–24].

To date, a synthesis of research on the effects of suicide bereavement on physical health problems and psychosomatic symptoms has not been conducted. The rationale for this review is to contribute to the evidence around the societal impact of suicide bereavement as borne by the families and health services, as well as informing clinicians who support those bereaved by suicide. The population of interest is bereaved family members and the exposure of interest is suicide bereavement. Therefore, people bereaved by suicide will be compared to people bereaved by other causes of death to examine any differences in physical and psychosomatic health between the two groups. The aim of this paper is to examine the physical and psychosomatic morbidities of people bereaved by a family member’s suicide compared with family members bereaved by other modes of death.

## Methods

This review was conducted by adhering to the Preferred Reporting Items for Systematic Reviews and Meta-Analyses (PRISMA) checklist [25]. The PRISMA checklist has been completed (see Additional file 1). The review protocol has been registered on PROSPERO, registration number CRD42016030007.

### Search strategy

MEDLINE, EMBASE, CINAHL, and PsycINFO were searched for articles published between 1^st^ January 1985 and 15^th^ February 2016. Searching was re-run on 27^th^ March 2017 to locate additional articles published in the interim (*n* = 666). This search found one recently published study that met the study inclusion criteria. The following MeSH terms were exploded to define exposure: “suicide”, “bereavement”, “genetic predisposition to disease” and “family characteristics”. Searches for the following keywords were also run to define exposure: “grief”, “familial”, “family history” and “genetic predisposition”. The following MeSH terms were exploded to define the population of interest: “family” and “friends”. The term “friends” was included in order to ensure inclusion of all relevant articles that may have included family members also. The term “survivors” was not exploded as it would have included survivors of terminal illness and long-term HIV survivors. Searches for the following keywords were also run to define the population: “relative*”, “parent*”, “mother*”, “father*”, “sibling*”, “offspring*”, “child*”, “brother*”, “sister*”, “family”, “friend” and “survivor*” (see Additional file 2). Searches were limited to English language articles only and articles published from 1985 to 15th February 2016 for the first search. As previously stated, the search was re-run in March 2017. There were slight modifications to this search strategy when searching other databases, where needed/appropriate. Reference list searching was employed for all included studies. The search strategies used for each of the databases is provided in Additional file 2. Among the full-texts of articles retrieved, sixty were subsequently excluded. The citations of these articles along with the reasons for their exclusion are provided in Additional file 3.

### Inclusion criteria

Studies that met the following eligibility criteria were included in the review: (1) the population of interest comprised family members bereaved by suicide, including those related by blood and also including spouses; (2) controls were family members bereaved by a non-suicide death; (3) authors specified at least one physical or psychosomatic health outcome; (4) original cross-sectional, case-control, cohort and registry-based studies.

### Exclusion criteria

Studies that exclusively used non-bereaved controls as the comparison group were excluded, as it is impossible to say if any negative health effect observed is attributable to suicide bereavement or to bereavement in general [4]. Case reports, cases studies, reviews, randomised controlled trials and studies with no control groups were excluded. If multiple articles meeting the inclusion criteria were published based on the same study, the article(s) containing the most complete or new information was used.

### Data collection and data extraction

The first author (AS) conducted the initial searches and screening process. Three authors (AS, CL and KMS) screened the full-text articles to assess for eligibility; disagreements were discussed and resolved with a fourth reviewer (EA). One author who was contacted regarding missing relevant information provided further analyses to meet the inclusion criteria for this review [26]. One author (AS) extracted the following information from relevant articles:

- Author and publication details
- Location/setting
- Study design
- Population/exposure/comparison group/outcome
- Methodological considerations (sample size, duration of participation and loss-to-follow-up)

### Quality assessment and analysis

A modified version of the Newcastle Ottawa Scale which was used in a previously published systematic review was chosen to assess risk of bias of individual studies at the study level [27]. Scores range from zero (high risk of bias) to three (low risk of bias). Definitions then follow in order to determine what constitutes low, moderate and high risk of bias. Two authors (AS and FR) independently assessed the quality of each included article. AS resolved any disagreements through discussion with another reviewer (EA). Articles were not excluded based on the quality assessment. Results of included studies were synthesised in narrative form.

## Results

### Search results

**Fig. 1:**
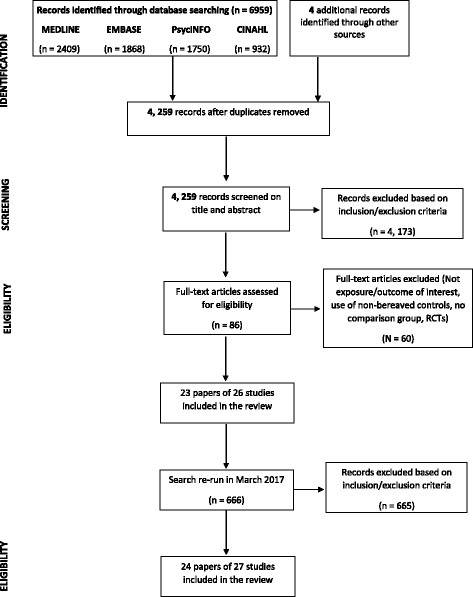
PRISMA flow diagram illustrating search process of systematic review

Figure 1 highlights the process of identifying relevant articles. A total of 6959 records were identified across the four databases, with four additional records identified from other sources, namely reference list searching. Eighty-six full-text articles were assessed for eligibility. The search was re-run in March 2017 which retrieved 666 articles that were published in the interim. One of these met the criteria for the study. Therefore, 24 papers meeting the inclusion criteria, representing 27 studies were included in the review. Three papers were published using the same study sample. Where this occurred, the most up-to-date or most comprehensive information and results were included. This was done to ensure that information was not duplicated in the review [21, 28, 29]. Of the 24 included studies, five studies examining aspects of physical health [19, 30–33] found that family members bereaved by suicide had statistically poorer health outcomes than the non-suicide bereaved comparison. Two further studies found statistically significant associations in the opposite direction, whereby the suicide-bereaved were at lower risk than the non-suicide bereaved comparison [34, 35]. No studies examining psychosomatic health outcomes found statistically significant results.

### Study characteristics

Table 1 outlines details of the 24 included studies. These were conducted in the United States (*n* = 9), Sweden (*n* = 4), Canada (*n* = 2), the Netherlands (*n* = 2), Denmark and Sweden (*n* = 1), Slovenia (*n* = 1), Denmark (*n* = 1), China (*n* = 1), Japan (*n* = 1), Norway (*n* = 1), and England (*n* = 1). The majority of the studies (13 studies) were conducted more than 10 years ago (1988–2003) [22, 24, 31, 33, 34, 36–43], with 11 studies published in the last ten years (2006–2017) [19, 20, 23, 26, 30, 32, 35, 44–47]. Included studies were 10 cross-sectional studies [23, 24, 30, 32, 33, 36, 38, 41–43], 11 cohort/registry-based studies [22, 26, 31, 34, 35, 37, 39, 44–47] and three case-control studies [19, 20, 40]. Eleven studies examined aspects of physical health, including general health, [19, 20, 26, 30, 31, 35, 39, 44–47], eight studies examined somatic complaints/reactions [24, 32, 33, 36, 37, 40, 42, 43], three studies examined psychosomatic health outcomes [22, 23, 41], while the remaining two studies examined both physical and psychosomatic health outcomes [34, 38]. Sample size varied considerably between studies ranging from 13 people bereaved by suicide to large-scale registry-based studies with 31,672 people bereaved by suicide. Length of follow-up was also diverse, ranging from nine months to 45 years.

**Table 1:** Study Characteristics and results

Study ID	Setting	Study design	Participants	Comparison	Outcome(s)	Results	Limitations of the study
Cerel et al., 1999 [34]	Ohio, United States	Cohort study Follow-up: 1, 6, 13 and 25 months after the death	*N* = 26 children bereaved by suicide of parent, from 15 families	*N* = 322 children bereaved by non-suicide death of parent (reasons other than suicide or homicide)	BAMO, an unvalidated scale measuring somatisation disorder Health/Sickness Questionnaire School and Physician Rating Forms	No significant difference in scores of somatisation between suicide and non-suicide bereaved. Suicide-bereaved children visited doctor less frequently but missed significantly more days of school than non-suicide-bereaved children	Small sample size for suicide-bereaved children. No confounding factors were controlled for. Type 1 error is increased due to multiple testing of the data
Cleiren et al., 1994 [37]	Leiden, The Netherlands	Cohort study Follow-up: 4 and 14 months after death	*N* = 91 first-degree relatives and spouses bereaved by suicide	*N* = 93 first-degree relatives and spouses bereaved by traffic accident *N* = 125 people bereaved by the illness of first-degree relative	General wellbeing questionnaire measuring physical health and somatic complaints (measure not described)	No differences were found for somatic complaints between the different modes of death groups (no *p*-value given). Mode of death was not significantly associated with physical health complaints	Some of the scales used are not validated. No confounding factors were controlled for. 10% loss to follow-up which may introduce attrition bias
Erlangsen et al., 2017 [35]	Denmark	Longitudinal cohort study Follow-up: 1980–2014	*N* = 15,607 suicide-bereaved spouses	*N* = 788,778 spouses bereaved by non-suicide death	Diagnosis of cancers, diabetes, sleep disorders, cardiovascular and chronic lower respiratory tract diseases, liver cirrhosis, and spinal disc herniation	Suicide-bereaved had lower risk of diagnoses of cancer, diabetes, cardiovascular and chronic lower respiratory tract disorders. They were less likely to take sick leave	Only people who were in a formal union or who were living together were included. While analyses was adjusted for some covariates, unmeasured confounders may be an issue
Fang et al., 2011 [44]	Sweden	Historic cohort study Follow-up: 1990–2004	*N* = 102 parents bereaved by suicide of child	*N* = 124 parents bereaved by non-suicide death of child *N* = 334 and *n* = 297 parents bereaved by non-cancer and cancer death of child *N* = 46 and *n* = 251 parents bereaved by an infection-related or any other cause of death of child	A diagnosis of infection-related cancer using ICD codes	The risk ratio was higher for suicide-bereaved than for non-suicide bereaved but this didn’t reach statistical significance; confidence intervals greatly overlapped	Some potential confounders were not accounted for due to the registry-based nature of the study
Farberow et al., 1992 [22]	Three counties in California	Cohort study Follow-up: 2, 6, 12 and 30 months after the death	*N* = 108 suicide-bereaved aged ≥55 years	*N* = 199 people aged ≥ 55 years bereaved by natural death *N* = 144 people aged ≥55 years not experiencing any death or divorce of spouse	The somatisation subscale of the Brief Symptom Inventory (BSI)	Suicide-bereaved and naturally bereaved spouses did not differ significantly on the somatisation subscale. All of the mean scores of the scales, including somatisation, decreased over the 2.5 year period	There appears to be loss to follow-up in each group which may indicate the presence of attrition bias
Grad and Zavasnik, 1999 [39]	Slovenia	Cohort study Follow-up: 2 and 12–14 months after the death	*N* = 30 suicide-bereaved spouses	*N* = 23 spouses bereaved by road traffic accident *N* = 20 spouses bereaved by terminal illness	Slovenia Bereavement Scale (SBS) has 46 items, representing several categories, including physical health	There were no statistically significant differences (no data presented) between the bereaved groups on the physical health measures contained within the SBS	Small sample size and no confounding factors controlled for. First assessment was conducted 2 months post-death when acute grief is likely to be present
Kennedy et al., 2014 [45]	Sweden	Historical cohort study Follow-up: 1961–2006	*N* = 19,535 offspring ≤18 years and *n* = 12,137 ≥ 18 years who lost a parent to suicide	*N* = 42,796 offspring ≤18 years and *n* = 136,786 ≥ 18 years bereaved by parental cancer death *N* = 52,592 offspring ≤18 years and *n* = 178,393 ≥ 18 years bereaved by parental non-cancer death *N* = 25,772 offspring ≤18 years and *n* = 18,566 ≥ 18 years bereaved by parental non-suicide death	Diagnosis of first malignant cancer before the age of 40 in the Cancer Register	The effect of suicide bereavement more than doubled the risk of human papillomavirus-related cancers before the age of 40, compared to those bereaved by non-suicide deaths. However, this finding was not statistically significant	No information on individual confounding factors including alcohol consumption and smoking
Momen et al., 2013 [26]	Denmark and Sweden	Population-based cohort study Follow-up for Denmark/ Sweden: 1968–2007/1973–2006	*N* = <66 suicide-bereaved (exact number unavailable from authors)	*N* = 1217 children bereaved by unexpected deaths, other than accident, suicide or the violence death of their relative	A diagnosis of childhood cancers using ICD codes	The adjusted hazard ratio was higher for suicide-bereaved children than children bereaved by other causes of death. However this association did not reach statistical significance	Small numbers of suicide- bereaved may not make these findings generalisable to other suicide-bereaved family members
Séguin et al., 1995 [31]	Quebec City, Montreal	Cohort study Follow-up: First interview M = 5.8 months after the death Second interview M = 9 months after the death	*N* = 30 parents who lost a son aged 18 to 35 years to suicide	*N* = 30 parents who lost a son aged 18 to 35 years by a car accident	Physical disorders were measured using items taken from Quebec’s 1987 Health Survey	Suicide-bereaved had more physical illnesses and consulted health professionals more frequently than accident survivors	Some important confounding factors including gender and age of the deceased not controlled for. High rate of attrition bias
Weinberg et al., 2013 [46]	United States	Prospective longitudinal controlled study Follow-up: 5 years	*N* = 45 offspring bereaved by suicide of a parent	*N* = 27 offspring bereaved by accidental death of a parent *N* = 51 offspring bereaved by sudden natural death of a parent	BMI was the outcome studied, by measuring the weight and height of offspring objectively	There were no differences in the BMI categories of offspring bereaved by suicide, accident and sudden natural death	Some participants recruited via advertising. Possible attrition bias as participants lost to follow-up more likely to be bereaved than those retained in the study
Wilcox et al., 2015 [47]	Sweden	Prospective cohort study Follow-up: 3 years	*N* = 537 parents bereaved by suicide of an offspring	*N* = 716 parents bereaved by accidental death of offspring *N* = 549 parents bereaved by natural death of offspring	Diagnosis-specific sickness absence exceeding 30 days due to somatic diagnoses	No statistically significant differences in the risk of somatic diagnosis between suicide-bereaved, accident-bereaved and naturally bereaved parents	Sickness absence due to specific somatic diagnoses were only included if they exceeded 30 days
Barrett and Scott, 1990 [36]	North Dakota and Minnesota, United States	Cross-sectional study	*N* = 14 suicide-bereaved spouses	*N* = 15 accident-bereaved spouses *N* = 15 unanticipated natural death bereaved *N* = 13 bereaved by theexpected natural death of spouse	Grief Experiences Questionnaire (GEQ): somatic reactions subscale	No significant differences in mean scores of somatic reactions for suicide-bereaved and non-suicide bereaved	Small sample size of suicide and non-suicide bereaved
De Groot et al., 2006 [30]	Northern Provinces in The Netherlands	Cross-sectional study	*N* = 153 first-degree suicide-bereaved relatives and spouses	*N* = 70 first-degree relatives and spouses bereaved by natural causes	RAND-36 used to assess general health, with nine subscales	Suicide-bereaved functioned less well in terms of pain and general health than naturally-bereaved	Possibility of selection bias due to difficulty in recruiting family members bereaved by natural death
Demi and Miles, 1988 [38]	United States	Cross-sectional study	*N* = 59 (15 fathers and 44 mothers) parents whose children died by suicide	*N* = 61 (13 fathers and 48 mothers) whose children died as a result of an accident or a chronic disease	Hopkins Symptom Checklist (HSCL) Health problems measured using the Bereavement Health Assessment Scale (BHAS)	No difference on the 5 subscales of the HSCL (somatisation, obsessive-compulsive, interpersonal sensitivity, depression, anxiety) or across physical health outcomes between the 2 groups	Bereaved parents may not be representative as they were recruited from self-help groups
Dyregrov et al., 2003 [33]	Norway	Cross-sectional study	*N* = 128 suicide-bereaved parents	*N* = 68 accident-bereaved parents *N* = 36 SIDS-bereaved parents	General Health Questionnaire (GHQ-28): somatic symptoms	SIDS-bereaved parents experienced significantly fewer problems on GHQ than suicide and accident-bereaved	Control group was heterogenous (violent and non-violent deaths)
Kitson, 2000 [41]	Two Midwestern counties in United States	Cross-sectional study	*N* = 85 suicide- bereaved widows	*N* = 56 homicide-bereaved widows *N* = 135 accident-bereaved widows *N* = 167 sudden death bereaved widows *N* = 106 long-term illness bereaved widows	The somatisation subscale of the Brief Symptom Inventory (BSI)	No differences between the 5 bereaved groups on somaticism	Control group contained both bereavement from violent and non-violent deaths which may have introduced selection bias
McNiel et al., 1988 [42]	United States	Cross-sectional study	*N* = 13 widows bereaved by the suicide death of their husband	*N* = 13 widows bereaved by the accidental death of their husband	General Health Questionnaire (GHQ): somatic complaints subscale	No significant differences in the mean scores of suicide and accident-bereaved	Very small sample size and no confounding factors were adjusted for
Miyabayashi and Yasuda, 2007 [32]	Japan	Cross-sectional study	*N* = 21 suicide-bereaved adults	*N* = 23 accident-bereaved adults *N* = 9 adults bereaved by acute illness *N* = 74 adults bereaved by shorter illness *N* = 88 adults bereaved by longer illness	General Health Questionnaire (GHQ), including somatic symptoms	No group differences were found for somatic symptoms. Multiple comparison tests indicated that those bereaved by suicide had poorer general heath than those bereaved by a longer illness (*p* < 0.05)	Selection bias may be present as participants recruited from self-help group. Response bias may be present due to the small sample of suicide-bereaved and those bereaved by acute illness. Some important confounders were not controlled for
Pfeffer et al., 2000 [24]	United States	Cross-sectional study	*N* = 11 families (made up of 16 children) where a parent died by suicide	*N* = 57 families (made up of 64 children) where a parent died from cancer	Child Behaviour Checklist (CBCL) has a subscale for somatic complaints	Mean scores of somatic complaints did not significantly differ between children bereaved by the cancer death of a parent and the suicide death of a parent	Some participants recruited via advertising which could lead to response bias. Very small sample of suicide-bereaved which will not be generalisable
Reed and Greenwald, 1991 [43]	United States	Cross-sectional study	*N* = 85 suicide-bereaved relatives and spouses	*N* = 96 accident-bereaved relatives and spouses	Measure for somatic complaints with 6-items	No significant differences in somatic complaints between the two groups	Use of unvalidated measures throughout the study
Xu and Li, 2014 [23]	China	Cross-sectional study	*N* = 92 immediate family members (parents, siblings, children, spouses) bereaved by suicide	*N* = 64 immediate family members (parents, siblings, children, spouses) bereaved by accidental death	The Symptom Checklist-90-Revised (SCL-90-R) has nine subscales, including somatisation	No significant differences were found on the score of somatisation between the suicide-bereaved group and the accidental death group	May not be generalisable to wider bereaved group as findings may be culturally specific
Bolton et al., 2013 [19]	Manitoba, Canada	Longitudinal case-control study	*N* = 1415 parents of children that died by suicide	*N* = 1132 parents of children who died in an motor vehicle crash *N* = 1415 non-bereaved parents	Physical health disorders based on ICD 9 and 10 codes Outpatient physician visits for physical health and hospitalisation for physical illnesses	Two years pre and post-death, suicide-bereaved parents had significantly higher rates of CVD COPD, hypertension, diabetes, and outpatient physician visits for physical illnesses compared to motor-vehicle bereaved parents	Prevalence of physical disorders were examined two years pre-death and two years post-death. This time may not be sufficient for the development of certain physical health problems
Harwood et al., 2002 [40]	England	Case-control study	*N* = 46 adults bereaved by the suicide of an older adult	*N* = 46 adults bereaved by the natural death of an older adult	Grief Experiences Questionnaire (GEQ): somatic reactions subscale	No significant difference on somatic reactions for suicide-bereaved and naturally-bereaved	Small sample size may have increased the risk of type II error
Huang et al., 2013 [20]	Sweden	Nested case-control study	*N* = 792 parents bereaved by the suicide of a child	*N* = 1451 bereaved by non-self-inflicted death of child *N* = 1066 bereaved by cancer of child *N* = 2814 bereaved by non-cancer death of child	Pancreatic cancer, identified by the Swedish Cancer Register	It appears that suicide-bereaved have a higher risk of cancer but this finding is not statistically significant when compared with non-suicide bereaved	Unmeasured potential confounders for pancreatic cancer, including smoking and BMI could not be controlled for.

### Risk of bias assessment

The modified version of the Newcastle Ottawa Scale used has nine questions relating to five domains of evaluation, namely: selection of study participants (selection bias); controlling for confounding (performance bias); statistical methods (detection bias), measuring outcome variables (information bias); and subject follow-up (only for follow-up studies; attrition bias). Following the assessment of included studies using the modified version of the Newcastle Ottawa Scale, a number of study limitations emerged. Firstly, some studies (4/24) recruited study participants by advertising or recruiting from self-help groups, which may have introduced selection bias into the studies. Nearly half (11/24) of all included studies had small sample sizes, with the smallest sample being 13 suicide-bereaved widows. This small sample size may have reduced the likelihood of being able to identify a statistically significant difference between the suicide-bereaved and non-suicide bereaved groups with respect to physical and psychosomatic health. Seven of the studies had suicide-bereaved sample sizes of 30 participants or less. Over a quarter (6/24) of included studies either did not control for any confounding factors (4/24) or only adjusted for limited confounding factors (2/24). A small minority of studies (4/24) controlled for various factors including pre-bereavement functioning, kinship, cause of death, decedent’s gender and age and time since death. Overall, statistical analysis conducted across the papers was good, with the use of appropriate statistical methods. However, it was noted that over a quarter of studies (6/24) carried out multiple testing that was not accounted for, had inconsistent or no reporting of *p*-values and 95% confidence interval thresholds, and data was not presented for some analyses that were conducted. Half of the studies (12/24) contained heterogeneous control groups, where family members bereaved by violent (accident, homicide) and non-violent deaths (natural anticipated, natural unanticipated) were analysed together. This may have introduced selection bias as research indicates that health consequences differ when the death is violent or nonviolent [17].

A comparison of characteristics of responders and non-responders was present in a minority of the studies (2/24), with the majority of papers not presenting this information (13/24). Six studies were register-based studies and therefore, the issue of non-response bias is not applicable. One study did not have any information on non-responders beyond gender, age, mode of death and place of residence of deceased, due to confidentiality reasons. A further study compared excluded cases to included cases on a number of variables including victim’s age, race, sex and method of death and concluded there was no evidence of sample bias. Finally, one study compared bereaved offspring that remained in the study to those lost to follow-up. Bereaved offspring lost to follow-up were more likely than those who remained in the study to have a caregiver with a history of alcohol or substance disorder (32.1% vs. 16.7%), to have a caregiver of minority status (28.4% vs. 11.7%), and to have had a proband with a history of an anxiety disorder (28.3% vs. 16.4%). Overall, selection bias emerged as an important methodological consideration in the included papers (Table 2).

**Table 2:** Risk of bias assessment using Modified Version of Newcastle Ottawa Scale

	Is the source population appropriate and representative of population of interest?	Is the source sample size sufficient and is there sufficient power to detect a meaningful difference in outcome?	Did the study adjust for any variables or confounders that may influence the outcome?	Did the study use appropriate statistical analysis methods relative to the outcome of interest?	Is there little missing data and did the study handle it accordingly?	Is the methodology of the outcome measurement explicitly stated and is it appropriate? Is there an objective assessment of outcome?	Was the follow-up sufficiently long enough for the outcome to occur?	Was there minimal loss to follow-up and are subjects lost to follow-up unlikely to introduce bias?
Barrett & Scott, (1990) [36]	2	0	0	2	3	2	NA	NA
Bolton et al., (2013) [19]	2	3	3	2	3	3	2	3
Cerel et al., (1999) [34]	2	1	0	2	0	3	3	1
Cleiren et al., (1994) [37]	2	2	0	1	3	1	2	2
Demi and Miles, (1988) [38]	0	2	1	2	3	2	NA	NA
De Groot et al., (2006) [30]	2	3	2	3	3	2	NA	NA
Dyregrov et al., (2003) [33]	3	3	2	3	3	2	NA	NA
Erlangsen et al., (2017) [35]	3	3	2	3	3	3	3	3
Fang et al., (2011) [44]	3	3	2	3	3	3	3	3
Farberow et al., (1992) [22]	2	3	3	3	3	2	2	1
Grad and Zavasnik, (1999) [39]	3	1	0	2	3	2	2	1
Harwood et al., (2002) [40]	2	1	2	2	3	2	NA	NA
Huang et al., (2013) [20]	2	3	2	3	3	3	3	3
Kennedy et al., (2014) [45]	3	3	2	3	3	3	3	3
Kitson, (2000) [41]	2	2	2	2	3	2	NA	NA
Miyabashi and Yasuda, (2007) [32]	0	1	1	2	2	2	NA	NA
Momen et al., (2013) [26]	3	1	3	3	3	3	3	3
McNeil et al., (1988) [42]	2	0	0	2	3	2	NA	NA
Pfeffer et al., (2000) [24]	1	0	2	2	2	2	NA	NA
Reed and Greenwald, (1991) [43]	3	2	2	2	3	1	NA	NA
Seguin et al., (1995) [31]	2	1	2	2	3	2	2	1
Weinberg et al., (2013) [46]	1	1	2	3	3	3	3	1
Wilcox et al., (2015) [47]	2	3	2	3	3	2	3	3
Xu and Li, (2014) [23]	3	2	3	2	3	2	NA	NA

### Suicide bereavement and general health

Seven studies examined general health. Two cross-sectional studies found significant associations between suicide bereavement and general health [30, 32]. The first study by De Groot and colleagues included a Dutch sample of 223 bereaved family members. Suicide-bereaved family members scored more negatively than family members bereaved by natural death on a number of domains of the RAND scale, including pain, general health and experiencing a change in health following the death when compared to those bereaved by a natural death immediately before the death [30]. When analyses were adjusted for demographics, neuroticism and expectedness of death, pain was the only health-related measure that remained significant (95% CI: −.7, −.003) [30]. Selection bias may be an issue in this study because just 45% of approached suicide-bereaved families took part in the study. The second study showed that the general health of those bereaved by suicide (*n* = 21) was significantly poorer than those bereaved by a long-term illness (*n* = 88) (*p* < 0.05) [32]. However, participants were recruited from self-help groups and seminars for the bereaved and thus represent a biased sample of bereaved individuals.

Two cohort studies found statistically significant associations between general health and suicide bereavement [31], with one study finding an inverse association [34]. The first study of 60 bereaved family members found that those bereaved by suicide (*n* = 30) reported “more physical illnesses” and greater frequencies of appointments with healthcare professionals than those bereaved by accidental death (*n* = 30) [31]. Length of follow-up was relatively short, with the first and second interview occurring a mean 5.8 (range 4–8) and 9 (range 7–11) months after the death. In addition, nearly one-third of the suicide-bereaved sample (30.6%) refused to take part in the follow-up interview which may have introduced attrition bias. The second cohort study consisted of 26 suicide-bereaved and 332 non-suicide bereaved children and adolescents conducted in the United States by Cerel and Colleagues. In contrast to Séguin’s findings, the suicide-bereaved youth had significantly fewer visits to a doctor at 13 months (mean ± SD = 0.7 ± 1.1 versus 2.0 ± 3.3; *t* = 2.71, df = 24.5, *p* < .05) and 25 months post-bereavement (mean ± SD = 1.8 ± 2.7 versus 6.0 ± 8.5; *t* = 3.50, df = 18.4, *p* < .005) [34]. Séguin and colleagues found that suicide-bereaved parents visited health professionals more frequently than accident-bereaved parents [31]. The study by Cerel and colleagues also found that school-reported health problems did not vary between the suicide and non-suicide bereaved groups. In addition, non-suicide-bereaved offspring had missed significantly more days from school than suicide-bereaved offspring (2.8 ± 3.8 versus 0.8 ± 0.8; *t* = 2.78, df = 10.1, *p* < .05). This study had a very small sample of suicide-bereaved (*n* = 26) in comparison to an over-representation of non-suicide bereaved (*n* = 332) participants. A greater number of suicide-bereaved participants would have been preferable in order to have more balanced exposure groups for comparison purposes.

Two further cohort studies conducted in Slovenia [39] and The Netherlands [37] failed to find any significant association between suicide bereavement and physical health. The first study had a small sample size of 30 suicide-bereaved, 23 accident bereaved, and 20 spouses bereaved by a long-term illness (no *p*-value reported) [39]. The second study included a sample of 309 people bereaved by the death of a first-degree relative at four and 14 months after the death, but only controlled for limited confounders including sex, kinship and mode of death [37]. Lastly, a cross-sectional study conducted in the United States by Demi and Miles failed to find a significant difference between 59 suicide and 61 non-suicide bereaved parents with regard to physical health problems [F(4, 95 = 1.52, *p* = .20] [38]. However, the suicide-bereaved sample was recruited from self-help groups through various means of contact, indicating that a response rate for the suicide-bereaved group could not be reported. In addition, since only parental age was controlled for in the analysis, other important confounding factors were not taken into account.

### Suicide bereavement and specific physical disorders

Eight studies examined specific physical disorders and suicide bereavement. Four cohort/register-based studies examined the possible association between various forms of cancer following suicide bereavement. One additional registry-based study examined a number of physical health conditions, including cancer, cardiovascular disease, diabetes and suicide bereavement [35].Two Swedish register-based studies concluded that, being bereaved by the suicide death of an offspring conferred a higher risk (RR: 1.24, 95% CI: 1.01–1.49) of infection-related cancers [44] and pancreatic cancer in parents [20] (OR: 1.23, 95% CI: 1.03–1.46) compared to those bereaved by a non-suicide death [44]. Nevertheless, these findings were not statistically significant when compared with non-suicide bereaved parents. Similarly, two other large national studies found no statistically significant association between loss of a parent due to suicide when compared to other unexpected causes of death with a maximum of 15 years and 40 years of follow-up, respectively [26, 45]. These four studies successfully met most of the quality assessment criteria [20, 44, 45] with the exception of one paper having a relatively small number of suicide-bereaved participants [26].

No significant differences were found in categories of BMI (normal, overweight, obese) by Weinberg and colleagues at the 5-year assessment in offspring bereaved by suicide (*n* = 45), accident (*n* = 27) or sudden natural death (*n* = 51) [46]. However, the sample size was relatively small with some participants being recruited via advertising. Using data provided in the paper by Wilcox and colleagues cohort study, additional calculations did not show any statistically significant differences in the risk of sickness absence due to somatic diagnosis between suicide-bereaved, accident-bereaved and naturally bereaved. [47]. This study met most of the quality assessment criteria with some minor limitations related to selection bias and outcome measurement.

A Canadian case-control study conducted by Bolton and colleagues found that suicide-bereaved parents (*n* = 1415) had a significantly increased risk of a number of specific physical health disorders both before and after their offspring’s death compared to 1132 accident-bereaved parents [19]. These include cardiovascular disease (2 years pre-death ARR: 1.54: 1.16–2.03; 2 years post-death ARR: 1.63: 1.23–2.16), hypertension (ARR 1.37: 1.19–1.59; ARR 1.32: 1.15–1.52), diabetes mellitus (ARR 1.45: 1.20–1.76; ARR 1.66: 1.37–2.00) and chronic obstructive pulmonary disease (ARR 1.68: 1.20–2.37; ARR 2.01: 1.40–2.90) [19]. In addition, suicide bereaved parents had an increased risk of visiting a physician for a physical illness (ARR 1.38: 1.15–1.65; ARR 1.39: 1.18–1.63) and also being hospitalised for a physical illness (ARR 1.49: 1.01–2.20; ARR 1.52: 1.07–2.16) [19]. This paper met most of the quality assessment criteria. In contrast, a Danish register-based study found that spouses bereaved by suicide (*n* = 15,607) had a lower risk of receiving a subsequent diagnosis of a number of physical health disorders compared to spouses bereaved by a non-suicide death (*n* = 788,778) [35]. These included cancers (men: IRR, 0.8; 95% CI, 0.7–0.9; women: IRR, 0.8; 95% CI, 0.7–0.9), diabetes (men: IRR, 0.6; 95% CI, 0.4–0.7; women: IRR, 0.6; 95% CI, 0.5–0.8), cardiovascular (men: IRR, 0.9; 95% CI 0.8–0.9; women: IRR, 0.9; 95% CI, 0.8–1.0), and chronic lower respiratory tract disorders (men: IRR, 0.8; 95% CI, 0.7–1.0; women: IRR, 0.7; 95% CI, 0.6–0.8). Suicide-bereaved were less likely to take sick leave (men: IRR, 0.8; 95% CI, 0.7–0.9; women: IRR, 0.8; 95% CI, 0.7–0.8), while men were less likely to visit a general practitioner than those bereaved by other causes of death (IRR, 0.9; 95% CI, 0.8–1.0). Also, suicide-bereaved women had lower use of somatic hospitals (IRR, 0.9; 95% CI, 0.8–1.0). Similarly, this study scored highly across all of the assessment domains [35].

### Suicide bereavement and physical symptoms/somatic complaints

Eight studies examined physical symptoms/somatic complaints. Three American cross-sectional studies found no significant difference in somatic complaints for suicide-bereaved and accident-bereaved widows [42] and next-of-kin [43] and suicide-bereaved and cancer-bereaved children [24]. Sample sizes for the suicide-bereaved were a particular issue for two of the studies with a sample of 13 [42] and 16 [24], respectively. A further cross-sectional study conducted in Norway found that parents bereaved by SIDS (Sudden Infant Death Syndrome) experienced significantly fewer problems (*p* < .05) than parents bereaved by suicide and accidents on the general health questionnaire (GHQ). Therefore, those bereaved by suicide and accident significantly differed from the SIDS sample with respect to their level of complaints on the GHQ (suicide: M = 9.8, SD = 8.3; accident: M = 10.4, SD = 7.8; vs. SIDS: M = 5.8, SD = 7.1, F = 4.17, *p* < .05) [33]. Cleiren and colleagues also found no significant difference in somatic complaints between 91 suicide-bereaved, 93 road traffic accident or 125 long-term-illness-bereaved first-degree relatives in a Dutch 10-month cohort study (data not presented in original paper) [37]. This study controlled for sex, kinship and mode of death only, which may have biased the results. Kinship, in this review refers to the type of familial relationship (parent-child, spousal, sibling, child-parent), including blood and non-blood relationships, between two people.

An American cross-sectional [36] and an English cohort study [40] found that somatic reactions did not significantly differ between suicide-bereaved and non-suicide bereaved participants. Specifically, the first study consisted of 14 suicide-bereaved (M = 12.86, SD = 4.57, *p* > .05), 15 accident-bereaved (M = 12.40, SD = 4.01, *p* > .05), 15 unanticipated naturally-bereaved (M = 12.67, SD = 3.27, *p* > .05) and 13 expected naturally bereaved widows/widowers (M = 11.08, SD = 3.01, *p* > .05) [36]. The second study included 20 suicide-bereaved and 18 naturally-bereaved children (M = 10.7 versus M = 9.9) [40]. Sample size was a significant limitation in both studies.

One final cross-sectional study conducted in Japan [32] did not find any significant group differences in somatic symptoms and complaints when comparing suicide-bereaved and non-suicide bereaved family members. The paper had hugely different sample sizes within the bereavement groups: suicide (*n* = 21), accidents (*n* = 23), acute illness (< 1 day) (*n* = 9), shorter illness (<1 year) (*n* = 74) or longer illness (≥ 1 year) (*n* = 88) [32]. This may have had an impact on identifying potential group differences.

### Suicide bereavement and somatisation

Five studies examined somatisation and suicide bereavement. Three were cross-sectional studies, two of which were conducted in the United States [38, 41], and one was conducted in China [23]. The American cross-sectional study conducted by Demi and Miles did not find significant differences between 59 suicide-bereaved and 61 non-suicide bereaved parents on the scale measuring distress, which included a somatisation measure [F (5, 111) = .45, *p* = .81] [38]. Participants were recruited via self-help groups and only parental age was adjusted for in the analysis. Similarly, the second American cross-sectional study concluded that mean scores on somatisation did not significantly differ between 85 suicide-bereaved (M = 9.9, SD = 9.9), 56 homicide-bereaved (M = 9.7, SD = 9.6), 135 accident-bereaved (M = 10.2, SD = 10.1), 167 sudden natural death bereaved (M = 9.7, SD = 9.7) and 106 long-term illness-bereaved widows (M = 10.8, SD = 10.8) [41]. The cross-sectional study conducted in China found no significant difference in somatisation between 92 suicide-bereaved and 64 accident-bereaved immediate family members (*p* = 0.87) [23]. Both of these studies met most of the quality assessment criteria. Cerel and colleagues conducted a cohort study in the United States which found no differences between 26 suicide-bereaved and 322 non-suicide bereaved children and adolescents with respect to somatisation [34]. Interviews were conducted with participants at 1, 6, 13 and 25 months post-parental death [34]. Sample size for the suicide-bereaved was a limitation in the study, together with the limitation that no confounding factors were adjusted for in the analysis. Similarly, the American cohort study conducted by Farberow and colleagues found that suicide-bereaved (*n* = 108) and naturally bereaved spouses (*n* = 199) did not differ significantly on the somatisation subscale (no *p*-value given) [22]. This study met most of the quality assessment criteria. However, it appears that there was a high rate of loss to follow-up in the study.

## Discussion

The current systematic review found 24 studies that fit the inclusion criteria. Of these, seven studies found statistically significant associations between aspects of physical health and suicide bereavement. Five studies noted that people bereaved by suicide had an increased risk of a number of adverse physical health outcomes. Two further studies found an association in the opposite direction for a number of physical health outcomes [35] and healthcare utilisation [34] for those bereaved by suicide.

This review of physical and psychosomatic health outcomes found tentative evidence supporting an association between bereavement by suicide and some physical health outcomes, although there are inconsistencies. Cardiovascular disease, COPD, hypertension, diabetes, increased pain and poorer general health were more frequently reported adverse physical health outcomes among people bereaved by suicide [19, 30–33] compared to those who experienced other types of bereavement. Some studies found that suicide bereavement conferred a lower risk of various physical and psychosomatic health outcomes [34, 35]. However, the majority of studies found no significant differences in physical and psychosomatic health outcomes following suicide bereavement [20, 22–24, 26, 36–47].

The prevalence of physical health issues in those bereaved by suicide [19, 30–33] may lead to more healthcare utilisation. There are varying findings with respect to healthcare utilisation amongst the suicide-bereaved. Suicide-bereaved adults were more likely to experience a health change after the death, have more appointments with healthcare professionals and also to be hospitalised more often for physical illnesses compared to non-suicide bereaved family members. In contrast, men bereaved by the suicide of a spouse were less likely to visit a general practitioner than those bereaved by other causes of death. In addition, both men and women bereaved by a spouse’s suicide were less likely to take sick leave than those bereaved by other causes of death [35]. Not seeking medical attention for physical health problems may be due to being preoccupied by grief [35, 48]. This underlies the importance for health care practitioners to be aware of the unique challenges of suicide bereavement and its associated health issues in their patients. Similarly, children bereaved by suicide missed significantly fewer days from school and also had fewer visits to a doctor compared to non-suicide bereaved children [34]. Suicide-bereaved children may have less familial environmental stressors and higher levels of functioning, including grief responses which have been shown to be important in moderating long-term outcomes for parentally bereaved children [16, 49]. Moreover, suicide-bereaved people experience more perceived stigma than those bereaved by both sudden unnatural and sudden natural death [50]. Shame and stigma have been linked to a number of avoidance behaviours, including poorer help-seeking in the suicide-bereaved [50–52]. Where people experience high levels of shame and stigma, this may impact negatively on their help-seeking behaviour which may in turn impact negatively on health outcomes. Additionally, a recent systematic review highlighted that stigma experienced by people bereaved by suicide was strongly correlated with increased somatic reactions, including headaches and stomach pain [51]. Therefore, it may also be plausible that shame and stigma may moderate the relationship between physical and psychosomatic health outcomes following suicide bereavement.

Following the synthesis of results, a number of issues associated with the included studies became apparent. Firstly, sample size was a significant limitation across a number of the studies, resulting in studies being underpowered, with some of the suicide-bereaved samples being as low as thirteen participants [42]. Some of the studies recruited participants from advertising, self-help and bereavement support groups, which biases the sample recruited as this group may be significantly different to those who do not attend support groups in terms of their own characteristics and grief responses [24, 32, 38, 46]. A number of studies did not adjust for any confounding factors [34, 36, 39, 42], and some adjusted only for a limited number of confounders including basic demographics of the deceased and/or surviving relative [32, 37, 38]. Only two of the included studies examined pre-bereavement physical health, which examined outcomes both before and after offspring death [19, 47]. Therefore, the majority of the studies included in this review only focus on changes to physical health after bereavement, and consequently, are subject to recall bias. Length of follow-up for the cohort/registry-based studies was generally considerable, with the shortest follow-up being nine months after the death. However, two of the studies conducted interviews with bereaved participants two and three months post-death. This short time span may bias results as acute grief reactions may still be present. Half of included studies had heterogeneous control groups, where both violent and nonviolent bereavements were included [20, 26, 32, 33, 36, 37, 39, 41, 44, 46, 47]. Research indicates that sense-making is significantly more challenging for people bereaved by violent deaths versus non-violent deaths [53]. In addition, those bereaved by suicide and drug-related death appear to be more affected by grief and mental health problems compared to those bereaved by accidental and natural deaths [54]. Therefore, the presence of heterogeneous control groups in these studies may underestimate the true impact of suicide bereavement on physical and psychosomatic outcomes.

Overall, the evidence to support an increased risk of adverse physical health outcomes following suicide bereavement is growing but further longitudinal controlled studies are needed. No study examining psychosomatic outcomes and suicide bereavement found a positive association. The use of objective measures of physical health is warranted in future studies, as much of the research conducted in this area have used self-reported measures of health which are subject to recall bias. Furthermore, more studies need to examine pre-bereavement physical health, which examines outcomes both before and after the death. Following on from this, uncertainty remains regarding psychosomatic health and suicide bereavement.

### Strengths and limitations

This is the first review to synthesise all relevant papers related to suicide bereavement and physical and psychosomatic health outcomes, using a rigorous, exhaustive and comprehensive search strategy. The PRISMA checklist guided the reporting of this review. This review also has some limitations. Firstly, only English-language studies published from 1985 to March 2017 were included. Only quantitative papers were included; differing results and conclusions may have been found with the additional inclusion of qualitative studies. It is possible that some differences in suicide bereavement may only be revealed through in-depth qualitative interviews as opposed to quantitative methods. The evidence indicates that suicide bereavement is associated with some adverse physical health outcomes, but there are inconsistencies across the studies. In addition, studies relating to psychosomatic health outcomes did not show an association with suicide bereavement. There was also an imbalance of studies reporting on physical health outcomes, with a small minority of papers solely focussing on psychosomatic health outcomes. This needs to be addressed in future research. Some of the papers investigating psychosomatic health outcomes had small sample sizes, selection bias and did not control for confounding factors. We need further research addressing the uncertainty regarding the association between physical and psychosomatic health outcomes and suicide bereavement as well as the specificity of these outcomes. Register-based and cohort studies are the most appropriate means of examining this research question; selecting an appropriate control group, people bereaved by sudden and violent deaths, is essential. Future studies also need to allow for sufficient time to follow-up as some of the outcomes may not be present shortly after bereavement.

## Conclusions

This systematic review found that a small number of studies demonstrated associations between suicide bereavement and adverse physical health outcomes, including cardiovascular disease, diabetes, chronic obstructive pulmonary disease, hypertension and poorer general health. However, most studies failed to conclude that people bereaved by suicide were at higher risk for a number of physical health conditions compared to non-suicide bereaved individuals. No studies found a significant association between suicide bereavement and psychosomatic health outcomes. Thus, the findings of this review indicate that, in terms of psychosomatic health issues at least, those bereaved by suicide may closely resemble people bereaved by other causes of death. Inconsistencies in results may be due to methodological shortcomings in the available studies, including inappropriate selection of control groups, small sample size and failure to control for confounding factors. Further longitudinal controlled studies need to be conducted in order to better understand the health implications of suicide bereavement, specifically compared to bereavement after sudden and violent deaths, including accident and homicide deaths.

## Additional files

Additional file 1: PRISMA checklist. (DOCX 16 kb)

Additional file 2: Search strategy. (DOCX 14 kb)

Additional file 3: List of excluded studies. (DOCX 62 kb)
