# Supplementary material for: Physical and psychosomatic health outcomes in people bereaved by suicide compared to people bereaved by other modes of death: a systematic review
Source: BMC Public Health. 2017 Dec 12;17:939. doi: 10.1186/s12889-017-4930-3 (PMC5725957; doi:10.1186/s12889-017-4930-3)
Supplement: Supplementary file 2 — Search strategy. (DOCX 14 kb) [file 12889_2017_4930_MOESM2_ESM.docx]

**Additional file 2: Search strategy**

**Search strategy: Medline, Platform: OVID (n = 2,409)**

1. exp Suicide/
2. suicid*.mp.
3. 1 or 2
4. exp Bereavement/
5. bereav*.mp.
6. grief.mp.
7. griev*.mp.
8. 4 or 5 or 6 or 7
9. exp Family/
10. family.mp.
11. exp Friends/
12. friend.mp.
13. (relative* or parent* or mother* or father* or sibling* or offspring* or child* or brother* or sister*).mp
14. Survivors/
15. Survivor*.mp.
16. 9 or 10 or 11 or 12 or 13 or 14 or 15
17. 3 and 8
18. exp Genetic Predisposition to Disease/
19. exp Family Characteristics/
20. familial.mp.
21. family history.mp.
22. genetic predisposition.mp.
23. 18 or 19 or 20 or 21 or 22
24. 3 and 23
25. 17 or 24
26. 16 and 25

**Search strategy: EMBASE, Platform: Elsevier (n = 1,868)**

1. ‘suicide’/exp
2. suicid*
3. #1 OR #2
4. ‘bereavement’/exp
5. bereav*
6. grief
7. griev*
8. #4 OR #5 OR #6 OR #7
9. ‘family’/exp
10. family
11. ‘friends’/exp
12. friend
13. relative* or parent* or mother* or father* or sibling* or offspring* or child* or brother* or sister*
14. ‘survivors’/de
15. survivor*
16. #9 OR #10 OR #11 OR #12 OR #13 OR #14 OR #15
17. #3 AND #8
18. ‘genetic predisposition’/exp
19. ‘family size’/exp
20. familial
21. ‘family history’
22. ‘genetic predisposition’
23. #18 OR #19 OR #20 OR #21 OR #22
24. #3 AND #23
25. #17 OR #24
26. #16 AND #25

**Search strategy: PsycINFO, Platform: EBSCO (n = 1,750)**

1. DE “Suicide”
2. SU suicid* OR TI suicid* OR AB suicid*
3. 1 OR 2
4. DE “Bereavement” OR DE “Grief”
5. SU bereav* OR TI bereav* OR AB bereav*
6. SU grief OR TI grief OR AB grief
7. SU griev* OR TI griev* OR AB griev*
8. 4 OR 5 OR 6 OR 7
9. DE “Family” OR DE “Biological Family” OR DE “Extended Family” OR DE “Family of Origin” OR DE “Interethnic Family” OR DE “Interracial Family” OR DE “Military Families” OR DE “Nuclear Family” OR DE “Schizophrenogenic Family” OR DE “Stepfamily”
10. SU family OR TI family OR AB family
11. SU friend* OR TI friend* OR AB friend*
12. SU (relative* or parent* or mother* or father* or sibling* or offspring* or child* or brother* or sister*) OR TI (relative* or parent* or mother* or father* or sibling* or offspring* or child* or brother* or sister*) OR AB (relative* or parent* or mother* or father* or sibling* or offspring* or child* or brother* or sister*)
13. DE “Survivors”
14. SU survivor* OR TI survivor* OR AB survivor*
15. 9 OR 10 OR 11 OR 12 OR 13 OR 14
16. 3 AND 8
17. SU genetic predisposition to disease OR TI genetic predisposition to disease OR AB genetic predisposition to disease
18. SU family characteristics OR TI family characteristics OR AB family characteristics
19. SU familial OR TI familial OR AB familial
20. SU family history OR TI family history OR AB family history
21. 17 OR 18 OR 19 OR 20
22. 3 AND 21
23. 16 AND 23
24. 15 AND 23

**Search strategy: CINAHL, Platform: EBSCO (n = 932)**

1. (MH “Suicide+”)
2. SU suicid* OR TI suicid* OR AB suicid*
3. 1 OR 2
4. (MH “Bereavement+”)
5. SU bereav* OR TI bereav* OR AB bereav*
6. SU grief OR TI grief OR AB grief
7. SU griev* OR TI griev* OR AB griev*
8. 4 OR 5 OR 6 OR 7
9. (MH “Family+”)
10. SU family OR TI family OR AB family
11. SU friend* OR TI friend* OR AB friend*
12. SU (relative* or parent* or mother* or father* or sibling* or offspring* or child* or brother* or sister*) OR TI (relative* or parent* or mother* or father* or sibling* or offspring* or child* or brother* or sister*) OR AB (relative* or parent* or mother* or father* or sibling* or offspring* or child* or brother* or sister*)
13. (MH “Survivors”)
14. SU survivor* OR TI survivor* OR AB survivor*
15. 9 OR 10 OR 11 OR 12 OR 13 OR 14
16. 3 AND 8
17. SU genetic predisposition to disease OR TI genetic predisposition to disease OR AB genetic predisposition to disease
18. (MH “Family Characteristics+”)
19. SU familial OR TI familial OR AB familial
20. SU family history OR TI family history OR AB family history
21. 17 OR 18 OR 19 OR 20
22. 3 AND 21
23. 16 AND 22
24. 15 AND 23
