# Supplementary material for: Physical and psychosomatic health outcomes in people bereaved by suicide compared to people bereaved by other modes of death: a systematic review
Source: BMC Public Health. 2017 Dec 12;17:939. doi: 10.1186/s12889-017-4930-3 (PMC5725957; doi:10.1186/s12889-017-4930-3)
Supplement: Supplementary file 3 — List of excluded studies. (DOCX 62 kb) [file 12889_2017_4930_MOESM3_ESM.docx]

**Additional file 3: List of excluded studies**

| Study ID | Reason for Exclusion |
| --- | --- |
| Agerbo et al[1] | Use of non-bereaved controls; not outcome of interest |
| Boelen et al[2] | Not exposure of interest |
| Bolton et al[3] | Results not presented separately for mode of death |
| Brent et al[4] | Use of non-bereaved controls; not outcome of interest |
| Brent et al[5] | Use of non-bereaved controls; not outcome of interest |
| Brent et al[6] | No comparison group; not outcome of interest |
| Brent et al[7] | Use of non-bereaved controls; not outcome of interest |
| Brent et al[8] | Not exposure of interest; not outcome of interest |
| Bron et al[9] | Not outcome of interest |
| Byrne and Raphael[10] | Use of non-bereaved controls; not exposure of interest |
| Cerel et al[11] | Not outcome of interest |
| Cerel et al[12] | No comparison group; not outcome of interest |
| Chen et al[13] | Not exposure of interest |
| Clarke and Wrigley[14] | Use of non-bereaved controls |
| Cleiren et al[15] | Not outcome of interest |
| Cho et al[16] | Use of non-bereaved controls |
| De Groot et al[17] | Not outcome of interest |
| Dyregrov and Dyregrov[18] | No comparison group |
| Erlangsen et al[19] | Not exposure of interest; not outcome of interest |
| Feigelman et al[20] | Not outcome of interest |
| Feigelman et al[21] | Not outcome of interest |
| Floyd et al[22] | Use of non-bereaved controls; not outcome of interest |
| Hamdan et al[23] | No comparison group; not outcome of interest |
| Hamdan et al[24] | No comparison group; not outcome of interest |
| Harrington et al[25] | Narrative review; not exposure of interest; not population of interest |
| Kovarsky[26] | Not outcome of interest |
| Levav et al[27] | Not exposure of interest |
| Kvikstad et al[28] | Results not presented separately for mode of death |
| Li et al[29] | Not exposure of interest; not outcome of interest |
| Li et al[30] | Results not presented separately for mode of death |
| Lohan and Murphy[31] | Not exposure of interest; not outcome of interest |
| McIntosh and Kelly[32] | Not outcome of interest |
| Melhem et al[33] | Not outcome of interest |
| Melhem et al[34] | Not outcome of interest |
| Melhem et al[35] | Not outcome of interest |
| Mitchell et al[36] | No comparison group |
| Mittendorfer-Rutz et al[37] | Use of non-bereaved controls; not exposure of interest |
| Muniz-Cohen et al[38] | Use of non-bereaved controls; not outcome of interest |
| Murphy et al[39] | Randomised Controlled Trial |
| Murphy et al[40] | Results not presented separately for mode of death |
| Murphy et al[41] | Results not presented separately for mode of death |
| Murphy et al[42] | Not outcome of interest |
| Murphy et al[43] | Results not presented separately for mode of death |
| Murphy et al[44] | Not outcome of interest |
| Murphy et al[45] | Randomised Controlled Trial |
| Pitman et al[46] | Not outcome of interest |
| Prigerson et al[47] | Not exposure of interest |
| Qin et al[48] | Not exposure or outcome of interest; use of non-bereaved controls |
| Range et al[49] | Not outcome of interest |
| Rostila et al[50] | Not outcome of interest |
| Rostila et al[51] | Not outcome of interest |
| Rubenowitz et al[52] | Use of non-bereaved controls |
| Saarinen et al[53] | No comparison group |
| Ségal[54] | Not outcome of interest |
| Séguin et al[55] | Not outcome of interest |
| Sorensen et al[56] | Not outcome of interest |
| Wolchik et al[57] | Not outcome of interest |
| Zetamer et al[58] | Not outcome of interest |
| Zisook and Lyons[59] | Results not presented separately for mode of death |
| Zisook and Shuchter[60] | Results not presented separately for mode of death |

1. Agerbo E: Midlife suicide risk, partner’s psychiatric illness, spouse and child bereavement by suicide or other modes of death: a gender specific study. *J Epidemiol Community Health* 2005, 59(5):407-412.

2. Boelen PA, Prigerson HG: The influence of symptoms of prolonged grief disorder, depression, and anxiety on quality of life among bereaved adults. *Eur Arch Psychiatry Clin Neurosci* 2007, 257(8):444-452.

3. Bolton JM, Au W, Chateau D, Walld R, Leslie WD, Enns J, Martens PJ, Katz LY, Logsetty S, Sareen J: Bereavement after sibling death: a population-based longitudinal case-control study. *World psychiatry : official journal of the World Psychiatric Association (WPA)* 2016, 15(1):59-66.

4. Brent DA, Moritz G, Bridge J, Perper J, Canobbio R: The impact of adolescent suicide on siblings and parents: a longitudinal follow-up. *Suicide Life Threat Behav* 1996, 26(3):253-259.

5. Brent DA, Bridge J, Johnson BA, Connolly J: Suicidal behavior runs in families. A controlled family study of adolescent suicide victims. *Arch Gen Psychiatry* 1996, 53(12):1145-1152.

6. Brent D, Melhem N, Donohoe MB, Walker M: The incidence and course of depression in bereaved youth 21 months after the loss of a parent to suicide, accident, or sudden natural death. *Am J Psychiatry* 2009, 166(7):786-794.

7. Brent DA, Melhem NM, Masten AS, Porta G, Payne MW: Longitudinal effects of parental bereavement on adolescent developmental competence. *J Clin Child Adolesc Psychol* 2012, 41(6):778-791.

8. Brent DA, Melhem NM, Oquendo M, Burke A, Birmaher B, Stanley B, Biernesser C, Keilp J, Kolko D, Ellis S: Familial Pathways to Early-Onset Suicide Attempt A 5.6-Year Prospective Study. *JAMA psychiatry* 2015, 72(2):160-168.

9. Bron B, Strack M, Rudolph G: Childhood experiences of loss and suicide attempts: significance in depressive states of major depressed and dysthymic or adjustment disordered patients. *J Affect Disord* 1991, 23(4):165-172.

10. Byrne GJ, Raphael B: The psychological symptoms of conjugal bereavement in elderly men over the first 13 months. *Int J Geriatr Psychiatry* 1997, 12(2):241-251.

11. Cerel J, Fristad MA, Weller EB, Weller RA: Suicide-bereaved children and adolescents: II. Parental and family functioning. *J Am Acad Child Adolesc Psychiatry* 2000, 39(4):437-444.

12. Cerel J, Maple M, Aldrich R, van de Venne J: Exposure to suicide and identification as survivor. Results from a random-digit dial survey. *Crisis: Journal of Crisis Intervention & Suicide* 2013, 34(6):413-419.

13. Chen JH, Bierhals AJ, Prigerson HG, Kasl SV, Mazure CM, Jacobs S: Gender differences in the effects of bereavement-related psychological distress in health outcomes. *Psychol Med* 1999, 29(02):367-380.

14. Clarke CS, Wrigley M: Suicide-related bereavement and psychiatric morbidity in the elderly. *Ir J Psychol Med* 2014, 21(1):22-24.

15. Cleiren MP, Grad O, Zavasnik A, Diekstra RF: Psychosocial impact of bereavement after suicide and fatal traffic accident: a comparative two-country study. *Acta Psychiatr Scand* 1996, 94(1):37-44.

16. Cho J, Jung SH, Kim C, Suh M, Choi YJ, Sohn J, Cho S-K, Suh I, Shin DC, Rexrode KM: Suicide loss, changes in medical care utilization, and hospitalization for cardiovascular disease and diabetes mellitus. *Eur Heart J* 2016, 37(9):764-770.

17. de Groot M, Kollen BJ: Course of bereavement over 8-10 years in first degree relatives and spouses of people who committed suicide: longitudinal community based cohort study, vol. 347; 2013.

18. Dyregrov K, Dyregrov A: Siblings After Suicide—“The Forgotten Bereaved”. *Suicide Life Threat Behav* 2005, 35(6):714-724.

19. Erlangsen A, Jeune B, Bille-Brahe U, Vaupel JW: Loss of partner and suicide risks among oldest old: a population-based register study. *Age Ageing* 2004, 33(4):378-383.

20. Feigelman W, Jordan JR, Gorman BS: Personal growth after a suicide loss: cross-sectional findings suggest growth after loss may be associated with better mental health among survivors. *Omega* 2009, 59(3):181-202.

21. Feigelman W, Jordan JR, Gorman BS: Parental grief after a child's drug death compared to other death causes: investigating a greatly neglected bereavement population. *Omega* 2011, 63(4):291-316.

22. Floyd FJ, Mailick Seltzer M, Greenberg JS, Song J: Parental bereavement during mid-to-later life: pre- to postbereavement functioning and intrapersonal resources for coping. *Psychol Aging* 2013, 28(2):402-413.

23. Hamdan S, Mazariegos D, Melhem NM, Porta G, Payne MW, Brent DA: Effect of parental bereavement on health risk behaviors in youth: a 3-year follow-up. *Arch Pediatr Adolesc Med* 2012, 166(3):216-223.

24. Hamdan S, Melhem NM, Porta G, Song MS, Brent DA: Alcohol and substance abuse in parentally bereaved youth. *J Clin Psychiatry* 2013, 74(8):828-833.

25. Harrington R, Harrison L: Unproven assumptions about the impact of bereavement on children. *J R Soc Med* 1999, 92(5):230-233.

26. Kovarsky RS: Loneliness and disturbed grief: a comparison of parents who lost a child to suicide or accidental death. *Arch Psychiatr Nurs* 1989, 3(2):86-96.

27. Levav I, Kohn R, Iscovich J, Abramson JH, Tsai WY, Vigdorovich D: Cancer incidence and survival following bereavement. *Am J Public Health* 2000, 90(10):1601-1607.

28. Kvikstad A, Vatten LJ: Risk and prognosis of cancer in middle-aged women who have experienced the death of a child. *Int J Cancer* 1996, 67(2):165-169.

29. Li J, Laursen TM, Precht DH, Olsen J, Mortensen PB: Hospitalization for mental illness among parents after the death of a child. *N Engl J Med* 2005, 352(12):1190-1196.

30. Li J, Johansen C, Bronnum-Hansen H, Stenager E, Koch-Henriksen N, Olsen J: The risk of multiple sclerosis in bereaved parents: A nationwide cohort study in Denmark. *Neurology* 2004, 62(5):726-729.

31. Lohan JAMSA: Bereaved Mothers' Marital Status and Family Functioning After a Child's Sudden, Violent Death: A Preliminary Study. *Journal of Loss & Trauma* 2007, 12(4):333-347.

32. McIntosh J, Kelly LD: Survivors' reactions: suicide vs. other causes. *Crisis* 1992, 13(2):82-93.

33. Melhem NM, Moritz G, Walker M, Shear MK, Brent D: Phenomenology and correlates of complicated grief in children and adolescents. *J Am Acad Child Adolesc Psychiatry* 2007, 46(4):493-499.

34. Melhem NM, Walker M, Moritz G, Brent DA: Antecedents and sequelae of sudden parental death in offspring and surviving caregivers. *Arch Pediatr Adolesc Med* 2008, 162(5):403-410.

35. Melhem NM, Porta G, Shamseddeen W, Walker Payne M, Brent DA: Grief in children and adolescents bereaved by sudden parental death. *Arch Gen Psychiatry* 2011, 68(9):911-919.

36. Mitchell AM, Sakraida TJ, Kim Y, Bullian L, Chiappetta L: Depression, Anxiety and Quality of Life in Suicide Survivors: A Comparison of Close and Distant Relationships. *Arch Psychiatr Nurs* 2009, 23(1):2-10.

37. Mittendorfer-Rutz E, Rasmussen F, Wasserman D: Familial clustering of suicidal behaviour and psychopathology in young suicide attempters. *Soc Psychiatry Psychiatr Epidemiol* 2008, 43(1):28-36.

38. Muniz-Cohen M, Melhem NM, Brent DA: Health risk behaviors in parentally bereaved youth. *Arch Pediatr Adolesc Med* 2010, 164(7):621-624.

39. Murphy SA: A bereavement intervention for parents following the sudden, violent deaths of their 12-28-year-old children: description and applications to clinical practice. *Can J Nurs Res* 1997, 29(4):51-72.

40. Murphy SA, Lohan J, Braun T, Johnson LC, Cain KC, Beaton RD: Parents' health, health care utilization, and health behaviors following the violent deaths of their 12- to 28-year-old children: a prospective longitudinal analysis. *Death Stud* 1999, 23(7):589-616.

41. Murphy SA, Braun T, Tillery L, Cain KC, Johnson LC, Beaton RD: PTSD Among Bereaved Parents Following the Violent Deaths of Their 12- to 28-Year-Old Children: A Longitudinal Prospective Analysis. *J Trauma Stress* 1999, 12(2):273-291.

42. Murphy SA, Clark Johnson L, Wu L, Fan JJ, Lohan J: Bereaved parents' outcomes 4 to 60 months after their children's deaths by accident, suicide, or homicide: A comparative study demonstrating differences. *Death Stud* 2003, 27(1):39-61.

43. Murphy SA, Johnson C, Lohan J: The Effectiveness of Coping Resources and Strategies Used by Bereaved Parents 1 and 5 Years after the Violent Deaths of Their Children. *OMEGA - Journal of Death and Dying* 2003, 47(1):25-44.

44. Murphy SA, Tapper VJ, Johnson LC, Lohan J: Suicide ideation among parents bereaved by the violent deaths of their children. *Issues Ment Health Nurs* 2003, 24(1):5-25.

45. Murphy SA, Johnson LC: Finding meaning in a child's violent death: a five-year prospective analysis of parents' personal narratives and empirical data. *Death Stud* 2003, 27(5):381-404.

46. Pitman AL, Osborn DPJ, Rantell K, King MB: Bereavement by suicide as a risk factor for suicide attempt: a cross-sectional national UK-wide study of 3432 young bereaved adults. *BMJ Open* 2016, 6(1).

47. Prigerson HG, Bierhals AJ, Kasl SV, Reynolds CF, 3rd, Shear MK, Day N, Beery LC, Newsom JT, Jacobs S: Traumatic grief as a risk factor for mental and physical morbidity. *The American Journal Of Psychiatry* 1997, 154(5):616-623.

48. Qin P, Mortensen PB: The impact of parental status on the risk of completed suicide. *Arch Gen Psychiatry* 2003, 60(8):797-802.

49. Range LM, Calhoun LG: Responses following Suicide and Other Types of Death: The Perspective of the Bereaved. *OMEGA - Journal of Death and Dying* 1990, 21(4):311-320.

50. Rostila M, Saarela J, Kawachi I: The Forgotten Griever: A Nationwide Follow-up Study of Mortality Subsequent to the Death of a Sibling. *Am J Epidemiol* 2012, 176(4):338-346.

51. Rostila M, Saarela J, Kawachi I, Hjern A: Testing the anniversary reaction: causal effects of bereavement in a nationwide follow-up study from Sweden. *Eur J Epidemiol* 2015, 30(3):239-247.

52. Rubenowitz E, Waern M, Wilhelmson K, Allebeck P: Life events and psychosocial factors in elderly suicides – a case–control study. *Psychol Med* 2001, 31(7):1193-1202.

53. Saarinen PI, Hintikka J, Viinamaki H, Lehtonen J, Lonnqvist J: Is it possible to adapt to the suicide of a close individual? Results of a 10-year prospective follow-up study. *Int J Soc Psychiatry* 2000, 46(3):182-190.

54. Segal NL: Suicidal Behaviors in Surviving Monozygotic and Dizygotic Co‐Twins: Is the Nature of the Co‐Twin's Cause of Death a Factor? *Suicide Life Threat Behav* 2009, 39(6):569-575.

55. Séguin M, Lesage A, Kiely M: History of Early Loss Among a Group of Suicide Survivors. *Crisis* 1995, 16(3):121-125.

56. Sorensen HJ, Mortensen EL, Wang AG, Juel K, Silverton L, Mednick SA: Suicide and mental illness in parents and risk of suicide in offspring: a birth cohort study. *Soc Psychiatry Psychiatr Epidemiol* 2009, 44(9):748-751.

57. Wolchik SA, Tein JY, Sandler IN, Ayers TS: Stressors, quality of the child-caregiver relationship, and children's mental health problems after parental death: the mediating role of self-system beliefs. *J Abnorm Child Psychol* 2006, 34(2):221-238.

58. Zetumer S, Young I, Shear MK, Skritskaya N, Lebowitz B, Simon N, Reynolds C, Mauro C, Zisook S: The impact of losing a child on the clinical presentation of complicated grief. *J Affect Disord* 2015, 170:15-21.

59. Zisook S, Lyons L: Bereavement and Unresolved Grief in Psychiatric Outpatients. *OMEGA - Journal of Death and Dying* 1990, 20(4):307-322.

60. Zisook S, Shuchter SR: Uncomplicated bereavement. *J Clin Psychiatry* 1993, 54(10):365-372.
